# Slope monitoring optimization considering three-dimensional deformation and failure characteristics using the strength reduction method: A case study

**DOI:** 10.1038/s41598-023-31249-9

**Published:** 2023-03-10

**Authors:** Jianxiu Wang, HubBoqiang Li, Yunhua Jiang, Puzhuo Tian, Ansheng Cao, Yanxia Long, Xiaotian Liu, Pengfei Si

**Affiliations:** 1 Key Laboratory of Geotechnical and Underground Engineering of the Ministry of Education, Tongji University, Shanghai, 200092 China; 2 Department of Geotechnical Engineering, Tongji University, Shanghai, 200092 China

**Keywords:** Civil engineering, Natural hazards

## Abstract

The potential deformation and failure of a slope with typical 3D shapes involve 3D characteristics, such that these factors cannot be simulated using 2D methods. If 3D characteristics are not considered in expressway slope monitoring, an excessive number of monitoring points may be arranged in the stable/safe part, whereas insufficient monitoring points may be arranged in the unstable/dangerous part. In this study, the 3D deformation and failure characteristics of the Lijiazhai slope of the Shicheng–Ji'an Expressway in Jiangxi Province, China were analyzed by 3D numerical simulations using the strength reduction method. The potential 3D slope surface displacement trends, initial position of failure, and maximum depth of potential slip surface were simulated and discussed. The deformation of Slope A was generally small. The slope ranging from the third platform to the slope top was located in Region I, where the deformation was approximately equal to zero. The deformation of Slope B was located in Region V, where the displacement generally was larger than 2 cm in the range from the first–third platforms to the slope top, and the deformation of the trailing edge exceeded 5 cm. The surface displacement monitoring points should be arranged in Region V. Monitoring was then optimized considering the 3D characteristics of the deformation and failure of a slope. Accordingly, surface and deep displacement monitoring networks were effectively arranged in the unstable/dangerous part of the slope. Results may be used as references for similar projects.

## Introduction

The potential deformation and failure of a slope with typical 3D shapes involve 3D characteristics. The lateral restraint and curvature of a 3D potential slip surface cannot be considered in 2D analysis, potentially resulting in conservative treatment and a huge waste of retaining structure. Monitoring is often adopted to ensure the safety of an expressway slope during the excavation and operation stages. For a slope with a typical 3D shape, arranging slope monitoring networks effectively is important. Geographic information system data acquisition, global positioning system communication, aerial technology, 3D laser scanning technology, and optical fiber sensing are widely used to obtain the 3D topographic characteristics of a slope. Modern digitization technologies such as digital photography and graphic analysis have been introduced into slope automatic monitoring. Moreover, 3D visual modeling technology and corresponding commercial software, such as interactive volume modeling, stratigraphic geo-cellular modeling, and geological object computer-aided design, have been developed to visualize the 3D shape of a slope. However, a visual model cannot provide a reference for the arrangement of the monitoring network of a slope. Slope monitoring networks are often arranged according to general provisions of specifications at equal distance, where the 3D characteristics of deformation and failure are not considered. For a slope with significant 3D deformation and failure features, an excessive number of monitoring points may be arranged in stable positions or an insufficient number of points may be arranged in unstable positions. Given that slope monitoring is costly, arranging the monitoring points in the appropriate monitoring positions is important.

In the early stages of research, the 3D characteristics of a slope were considered in numerical simulations. The effects of horizontal drains on groundwater level during rainfall were predicted using 3D finite element analyses of transient water flow through unsaturated and saturated soils for a slope^1^. A 3D failure surface was generated by elliptical lines based on the slip surface in the neutral plane and then extended in the Z direction, and a computer program called EMU-3D was coded to perform the calculation for practical problems^2,3^. A practical method of using non-uniform rational basis spline and ellipsoidal surfaces to simulate a 3D sliding surface was introduced^4^. Numerical finite element upper and lower bound limit analyses were used to produce stability charts for 3D homogeneous and inhomogeneous undrained slopes^5^. Based on the observed sliding block mechanism, a 3D analysis model was established that enabled variations in material strength mobilization within the sliding mass and at the slip surface of the Kettleman landfill slope. These studies showed slightly higher computed 3D factors of safety than the associated 2D values in pre- and post-slide cases. The failure mechanisms of 2D and 3D slopes were investigated by using the strength reduction method (SRM)^6^. This extensive study on the 3D effect was conducted with respect to boundary conditions, shear strength, and concentrated surcharge load. The results obtained by 2D and 3D analyses were compared, and the applicable scope of 2D and 3D methods was analyzed^7^. The 3D finite difference models were used in the analysis of slope failure in a lignite mine and indicated that the mobilized friction angle during major slope failure was significantly lower than the friction angle generated by the 2D limit equilibrium method (LEM)^8^. A residual soil slope in Singapore that failed on two occasions, namely, 1989 and 1991, was analyzed using 2D and 3D slope stability analyses. The results showed that the average shear strength parameters of the residual soils are representative of the slope and that the factors of safety (FOS) obtained from 2D slope stability analyses are not necessarily more conservative than those obtained from 3D slope stability analyses^9^. The reliability of cohesive soil slopes was assessed using a 3D probabilistic stability analysis algorithm. The effect of model parameters, including expected value, variance, and correlation distance of soil shear strength, on the reliability associated with particular failure mechanisms, was investigated^10^. A probabilistic approach was proposed using a modified version of the SAMU_3D model. This 3D slope stability software was used to account for complex geometry and was recently developed^11^. In several cases, 3D slope stability analysis was performed using SRM and LEM. The FOS and failure modes obtained by these two methods were generally in good agreement^12^. A 3D non-spherical critical slip surface that is more consistent with the actual slip surface in nature was obtained by using the 3d alternating variable local gradient optimization method^13^.

Numerous slope failures worldwide cause great financial loss and interruption of transportation. In extreme circumstances, slope failures may lead to loss of human lives. Proper analysis and design of a slope are essential to prevent such failures^14^. SRM has been widely used in slope stability analysis^15–17^. Recently, many slope analysis methods based on SRM have been developed. A virtual element method strength reduction technique for slope stability analysis was proposed by combining the SRM with the phi–nu inequality^18^. A novel hierarchical multiscale strength reduction method with a coupled finite element method (FEM) and discrete element method approach was proposed for a heterogeneous slope^19^. A stability analysis approach for fractured rock mass slopes was proposed by combining the SRM with undirected graph theory^20^. Using the extended FEM as a tool and based on the new definition, a new strength reduction numerical method to analyze the stability of a fractured rock slope was proposed^21^. For conventional strength reduction techniques that cannot be directly applied to geotechnical stability analyses involving nonlinear failure criteria, a generalized strength reduction concept was proposed to solve the geotechnical problems involving nonlinear failure models^22^. A new criterion considering the residual displacement increment criterion based on the displacement catastrophe criterion of characteristic points was presented^23^. A new nonlinear SRM based on the generalized Hoek–Brown criterion was presented to provide a reduction strategy with precise physical meaning and find an optimal set of parameters that trigger rock slope failure^23^. SRM was used to analyze the advantages of geotechnical slope stability, and the established FEM was solved using the SRM to obtain an accurate geotechnical slope stability coefficient and complete the accurate analysis of geotechnical slope stability^14^. A numerical model of 3D ancient landslide stability analyses was established by the local SRM and the SRM^24^. A material point SRM was used to investigate the stability of soil–rock mixture slopes and the whole process of large deformation occurring after destabilization^25^. Digital image processing technology was employed to establish the structure model of a real soil–rock mixture slope. Based on the structure model, the most recently developed strength reduction numerical manifold method was adopted to investigate the stability of the SRM slope^26^. An improved SRM that adopts the variable modulus elasto-plastic model to analyze the stability and deformation of the slope was developed^27^. The SRM is becoming increasingly popular in the stability analysis of slopes. Nevertheless, the criterion for slope failure associated with SRM is controversial, and divergence occurs while approaching the limit equilibrium state of slopes^28^. To avoid human error in these criteria and find a unified mechanical explanation for slope instability, a variational criterion was proposed^29^. A 3D slope model was constructed by extending the 2D model longitudinally to study the effects of the dilatancy angle using the PLAXIS 3D FEM with a built-in strength reduction technique^30^. The 3D stability of geocell-reinforced slopes was investigated using SRM where both the geocells and their infill and surrounding soils are taken into account^31^. A stability analysis of a selected Himalayan road cut slope from two different sections named sections (A) and (B) was performed, and the strength reduction factor based on the FEM was used to simulate the slope sections using Phase2 software^32^. A theoretical framework for monitoring the stability of a slope in an efficient and cost-effective manner was suggested, and the optimal arrangement of monitoring points was achieved using strength reduction finite element analysis and redundancy optimization analysis^33^. The stability of a typical 3D large-scale translational landslide was assessed by applying different methods and comparing the differences between them, and a typical large-scale translational landslide was modeled based on an actual project^34^.

In summary, these studies mainly focus on the new SRM and 3D mechanical analysis of a slope or a landslide during the excavation stage, but they seldom discuss the monitoring optimization of an operation slope considering 3D deformation and failure characteristics. In this manuscript, the Lijiazhai slope was selected as the background and the SRM was introduced to analyze the 3D slope mechanical characteristic during the operation stage. The displacement characteristics and plastic zone distribution obtained by SRM indicated the potential failure mode of the slope. The results were used to optimize the monitoring arrangement considering the 3D characteristic, which can provide a reference for similar engineering research.

## Background

The Shicheng–Ji'an Expressway is located in Jiangxi Province, China. This two-way, four-lane expressway is approximately 190 km in length, starting from Wuliting Shicheng through Ningdu, Meijiao, Dinglong, Guanxi, Taihe, and ending in Shitoushan in Ji'an. The Lijiazhai slope is located between the kilometer markers K92 + 660 and K92 + 825 and was formerly designed as a tunnel. The maximum designed excavation depth and height are 43.2 and 52.30 m. The slope is excavated in five steps, the slope ratios of which are 1:0.75, 1:1, 1:2.5, 1:1.5, and 1:2. The first step is 6 m high, the second to fourth steps are 8 m high, and the fifth step reaches the slope top. The step width is 2 m.

### Topography

**Figure 1:**
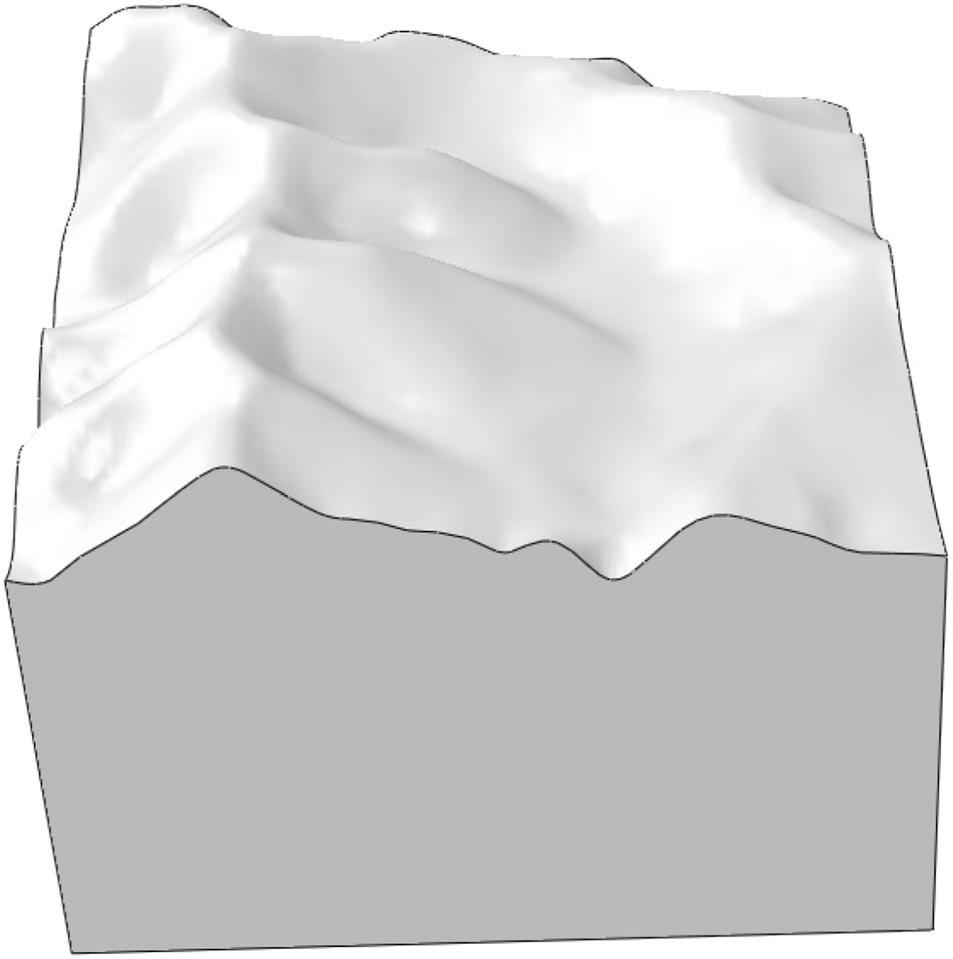
Original terrain of Lijiazhai slope before excavation.

The Lijiazhai slope is located in low mountain geomorphic units, including hillside, pass, erosion ditch, and alluvial valley (Fig. 1). The erosion ditches and alluvial valleys are developed. The strike directions of the erosion ditches are mainly northwest, north, and nearly north–south. The terrain is undulating. The elevation of the slope is between 238.4 and 304.3 m, and the relative elevation is approximately 65.90 m. The slope is steep, and the slope angles are between 30° and 35°. The slope axis is nearly orthogonal to the terrain contour.

### Formation of lithology

Quaternary Holocene slope residual sediments ($Q_{4}^{dl + el}$) are distributed at the two sides of the slope and on the hillside surface, which is mainly clay with sand fragments. Bedrock is exposed in the other positions and consists of Lower Sinian metamorphic sandstone ($Z_{1}$).

#### Quaternary Holocene slope residual sediments ($Q_{4}^{dl + el}$)

$Q_{4}^{dl + el}$ is yellow–brown, loam-based, and is in the plastic to hard plastic state. This layer is sandwiched between sand and gravel and distributed in the gully and some gentle passes. The maximum thickness of $Q_{4}^{dl + el}$ is approximately 6.0 m.

#### Sinian metamorphic siltstone ($Z_{1}$)

The palimpsest siltstone is gray-yellow to gray, has a palimpsest silty, thin-to-thick layered structure, and is composed of quartz, feldspar, and mica, which are fractured and strongly weathered. Phyllite is sandwich-like. Based on the degree of weathering, the layer can be divided into fully, strongly, and slightly weathered sub-layers.

The weakly weathered palimpsest siltstone has a dry compressive strength R_D_ = 8.8 MPa and a saturated compressive strength Rw = 5.3 MPa, C = 5.2 MPa, φ = 38.5°. The seismic velocities of the rock mass are shown in Table 1.

**Table 1:** Seismic velocity of rock mass.

Degree of weathering	Seismic velocity Vp (m/s)		
	Strong weathered	Weathered	Slightly weathered
Palimpsest siltstone phyllite	760–1170	1440–2190	3080–3970

### Geological structure

The Lijiazhai slope is located in a steadily expanding area in the middle-south Jiangxi Province, belonging to the western margin of the Wuyi uplift zone, Ganzhongnan folds (II2), South China fold system (I2). The strike direction of the strata is 90°∠70°. No active deep fault venue development exists in this area. Six groups of joints are developed, the strike directions of which are 300°∠48°, 2–3/m; 220°∠45°, 3–5/m; 360°∠68°, 5–6/m; 170°∠80°, 8–10/m; 290°∠50°, 1–2/m; and 250°∠12°, 2–3/m.

### Surrounding rock classification

The basic quality (BQ) of the rock in the expressway slope was determined based on design specifications^35^. The correction value [BQ] calculation formula is BQ = 90 + 3 Rc + 250 kV; Kv = (Vpm/Vpr)^2^.

Based on the surrounding rock BQ indicator, the slope rock quality indicators BQ and correction value [BQ] are shown in Table 2. The surrounding rock quality index correction.

**Table 2:** Rock mass quality indicators BQ and correction value [BQ].

Surrounding rock	Uniaxial compressive strength (MPa)	Integrity coefficient (Kv)	BQ	K1	K2	[BQ]
Slightly weathered Palimpsest siltstone phyllite	13	0.55	266.5	0.2	0.4	206.5

*Rw* rock uniaxial compressive strength (MPa) (average of the slightly weathered palimpsest siltstone and phyllite), *Kv* rock integrity coefficient, *K1* groundwater influence coefficient, *K2* weak structural plane occurrence affect the correction factor, *V*_pm_ the mass elastic longitudinal wave velocity (km/s), *V*_pr_ rock elastic longitudinal wave velocity (km/s).

Value calculation formula is [BQ] = BQ − 100 (K1 + K2).

The rock quality designation (RQD) values are shown in Table 3. The RQD svalue of the rock and other factors were obtained based on road slope design specification^36^, as well as on the degree of weathering of the rock mass, structural impact, structural features, and jointed degree of development in reference to the seismic velocity of rock mass. The characteristics and classification of the surrounding rock are shown in Table 4.

**Table 3:** Static of RQD.

Rock name	RQD (%)	Rock name	RQD (%)
Strongly weathered palimpsest siltstone	0–5.00	Slightly weathered palimpsest siltstone	8.00–28.00

**Table 4:** Rock characteristics and classification.

Location	Mileage	Length (m)	Surrounding rock	Physical and mechanical indicators	Integrate coefficient Kv, K1, K2	[BQ]	Integrity assessment	Surrounding rock classification
Left	ZK92 + 655 − ZK92 + 750	125	Residual slope, weathering palimpsest of strongly weathered siltstone section fissure development with fragmented structure, poor interlayer bonding	$$\begin{gathered} [\sigma_{0} ] = 800{\text{kPa}} \hfill \\ C = 7.5{\text{MPa}} \hfill \\ \varphi = 37.5^{ \circ } \hfill \\ \gamma = 18{\text{kN/m}}^{{3}} \hfill \\ K = 150{\text{MPa/m}} \hfill \\ E = 1.6{\text{GPa}} \hfill \\ \mu = 0.40 \hfill \\ \end{gathered}$$			Broken	V
	ZK92 + 790 − ZK92 + 820							
	ZK92 + 750 − ZK92 + 790	40	Slightly weathered palimpsest siltstone thick layered structure, block- (broken stone) (stone) like inlaid, fragmented structure, layered combination in general	$$\begin{gathered} Rw = 13{\text{MPa}} \hfill \\ [\sigma_{0} ] = 2000{\text{kPa}} \hfill \\ C = 15.2{\text{MPa}} \hfill \\ \varphi = 38.5^{ \circ } \hfill \\ \gamma = 20{\text{kN/m}}^{{3}} \hfill \\ K = 350{\text{MPa/m}} \hfill \\ E = 4.5G{\text{Pa}} \hfill \\ \mu = 0.30 \hfill \\ \end{gathered}$$	Kv = 0.55	206.50	Broken	IV
					K1 = 0.20			
					K2 = 0.40			

## Numerical model considering 3D deformation and failure characteristics using SRM

### Yield surface

**Figure 2:**
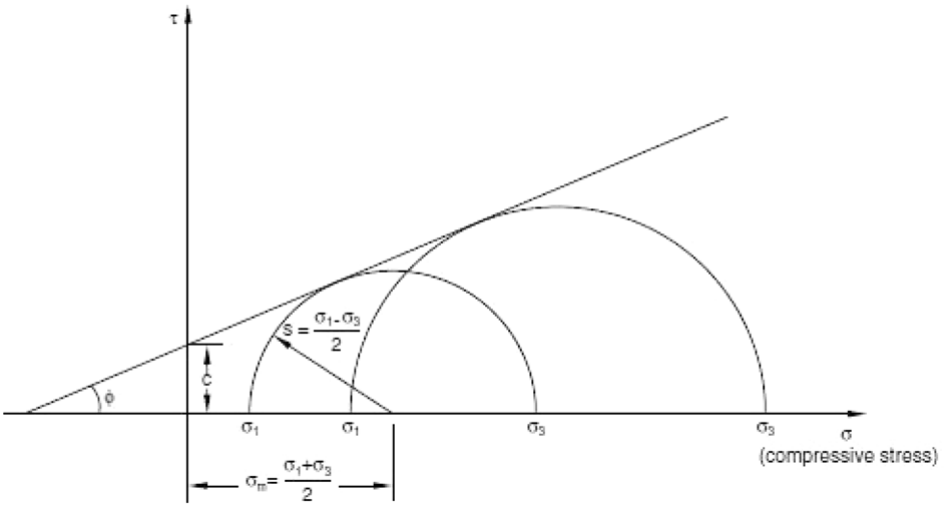
Mohr–Coulomb strength theory.

The Mohr–Coulomb plasticity model^37^ was adopted in the numerical simulations (Fig. 2).

The Mohr–Coulomb failure criterion can be expressed as

1 $$\tau = c - \sigma \tan \phi$$

where compression is negative.

The shear and normal stress of the failure point can be expressed as

2 $$\begin{array}{*{20}c} {\tau = s\cos \phi } & {\sigma = \sigma_{m} + s\sin \phi } \\ \end{array}$$

The following equation can then be obtained:

3 $$s + \sigma_{m} \sin \phi - c\cos \phi = 0$$

where $$s = \frac{1}{2}\,\left( {\sigma_{1} - \sigma_{3} } \right)$$;$$\sigma_{m} = \frac{1}{2}\,\left( {\sigma_{1} + \sigma_{3} } \right)$$.

**Figure 3:**
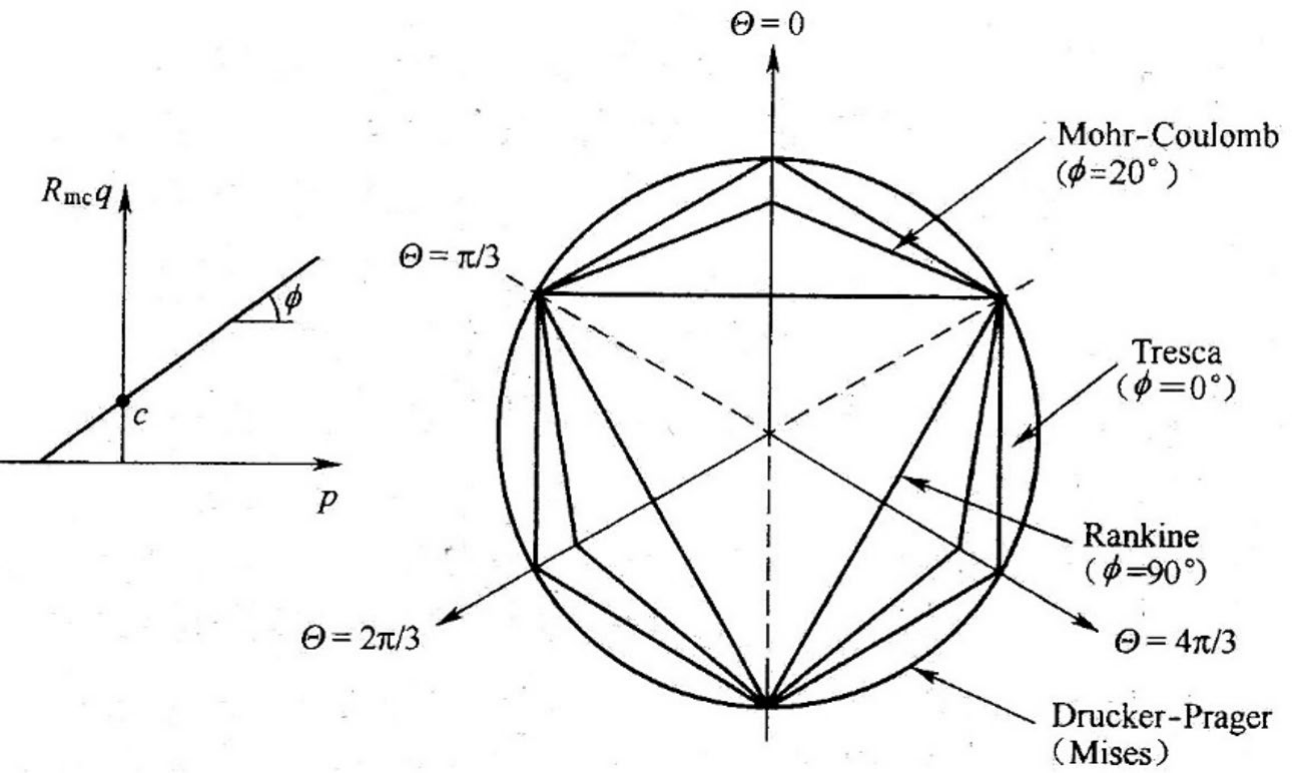
Yield surface of Mohr–Coulomb strength theory.

Equation (3) can be considered stress invariant to obtain the Mohr–Coulomb yield surface in the plasticity model (Fig. 3).

4 $$f = qR_{mc} - p\tan \phi - c = 0$$

where $\phi$ denotes the angle of friction of the material, i.e., the angle of the Mohr–Coulomb yield surface in the p–q stress plane (°); and $c$ denotes the cohesion (MPa). Function $R_{mc} \,\left( {\Theta ,\phi } \right)$ controls the shape of the yield surface in the $\pi$ plane and is calculated as follows:

5 $$R_{mc} \,\left( {\Theta ,\phi } \right)\, = \,\frac{1}{\sqrt 3 \cos \phi }\,\sin \left( {\Theta + \frac{\pi }{3}} \right) + \frac{1}{3}\,\cos \left( {\Theta + \frac{\pi }{3}} \right)\tan \phi$$

where $\Theta$ denotes the polar angle, $\cos \left( {3\Theta } \right) = \,\left( \frac{r}{q} \right)^{3}$;$p = - \frac{1}{3}trace\left( {\vec{\sigma }} \right)$;$p$ denotes the average stress or equivalent; $q$ denotes partial stress or Von Mises stress, $q = \sqrt {\frac{3}{2}\,\left( {\vec{S}:\vec{S}} \right)}$; $r$ denotes third partial stress invariant, $r = \,\left( {\frac{9}{2}\vec{S} \cdot \vec{S}:\vec{S}} \right)^{\frac{1}{3}}$; and $\vec{S}$ denotes the partial stress, $\vec{S} = \vec{\sigma } + p\vec{I}$.

### Flow potential

**Figure 4:**
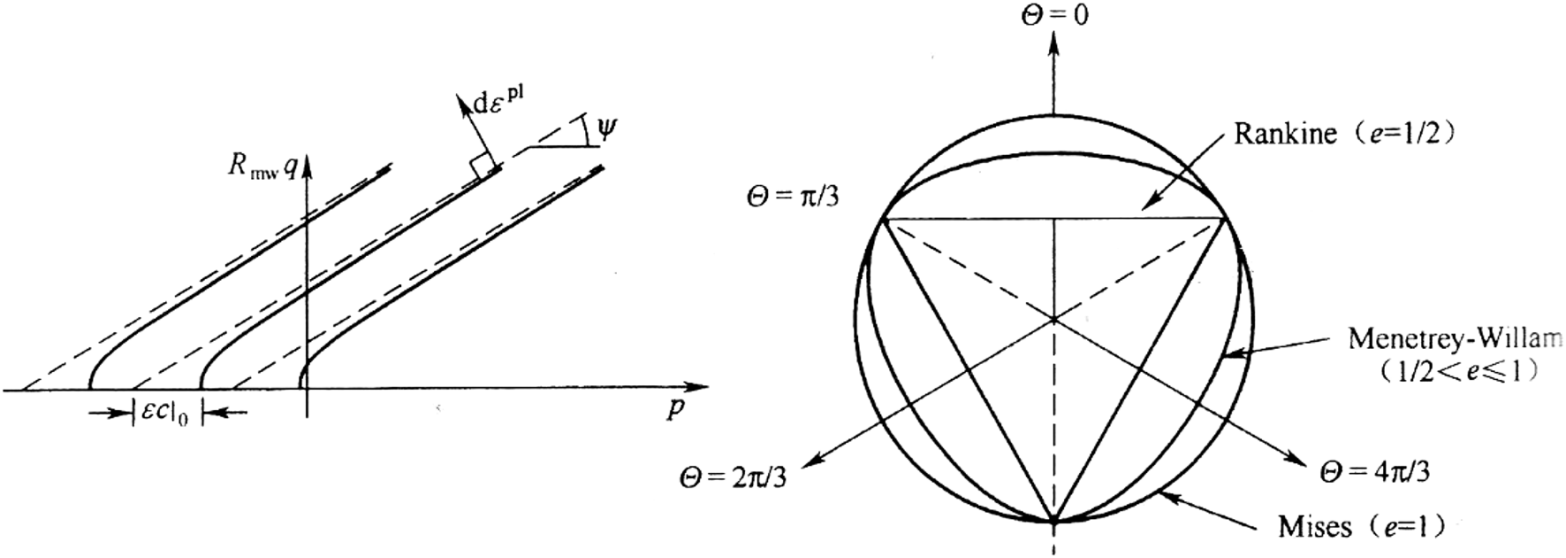
Flow potential of Mohr–Coulomb strength theory.

The current potential function presents a smooth oval shape (Fig. 4):

6 $$G = \sqrt {\left( {\zeta c{|}_{0} \tan \psi } \right)^{2} + \left( {R_{mw} q} \right)^{2} } \, - \,p\tan \psi ,$$

where $c{|}_{0} = c{|}_{{\overline{\varepsilon }^{pl} = 0}}$ denotes initial cohesion (MPa), and $\psi$ denotes the dilatancy angle (°). The eccentricity of the meridian plane, which controls the degree of similarity between the shape and function of the meridional plane asymptote function, likewise controls the shape in the plane. $R_{mw} \,\left( {\Theta ,e,\phi } \right)$ controls the shape of the meridian plane in the $\pi$ plane and is expressed as

7 $$R_{mw} \,\left( {\Theta ,e,\phi } \right) = \frac{{4\left( {1 - e^{2} } \right)\cos^{2} \Theta { + }\,\left( {2e - 1} \right)^{2} }}{{2\left( {1 - e^{2} } \right)\cos \Theta + \,\left( {2e - 1} \right)\,\sqrt {4\left( {1 - e^{2} } \right)\cos^{2} \Theta + 5e^{2} - 4e} }}R_{mc} \,\left( {\frac{\pi }{3},\phi } \right)$$

8 $$R_{mc} \,\left( {\frac{\pi }{3},\phi } \right) = \frac{3 - \sin \phi }{{6\cos \phi }},$$

where $e$ denotes surface eccentricity in the $\pi$ plane.

9 $$e = \frac{3 - \sin \phi }{{3 + \sin \phi }}$$

### Strength reduction

The slope safety factor is defined as (Bishop, 1955)

10 $$F = \frac{{\tau_{f} }}{\tau }$$

where $\tau_{f}$ denotes the shear strength of the sliding surface and $\tau$ denotes the sliding surface shear stress. In the actual analysis, the sliding of the slope along a slip surface is assumed. To determine whether the shear strength of the sliding surface satisfies the Mohr–Coulomb strength criterion, the following calculation is made:

11 $$\tau_{f} = c + \sigma \tan \phi$$

The reductions in the strength parameters of rock and soil $\left( {c,\,\phi } \right)$ are equal to the current safety reserve to maintain stability, in which the reduction parameters $c_{r} = \frac{c}{F}$,$\varphi_{r} = \frac{\tan \varphi }{F}$.

When Eq. (11) is satisfied for certain $c_{r}$ and $\varphi_{r}$ corresponds to *F*, plastic deformation appears. When the plastic units are connected and a penetrating plastic surface is formed, the slope loses stability.

### Numerical modeling stages

**Figure 5:**
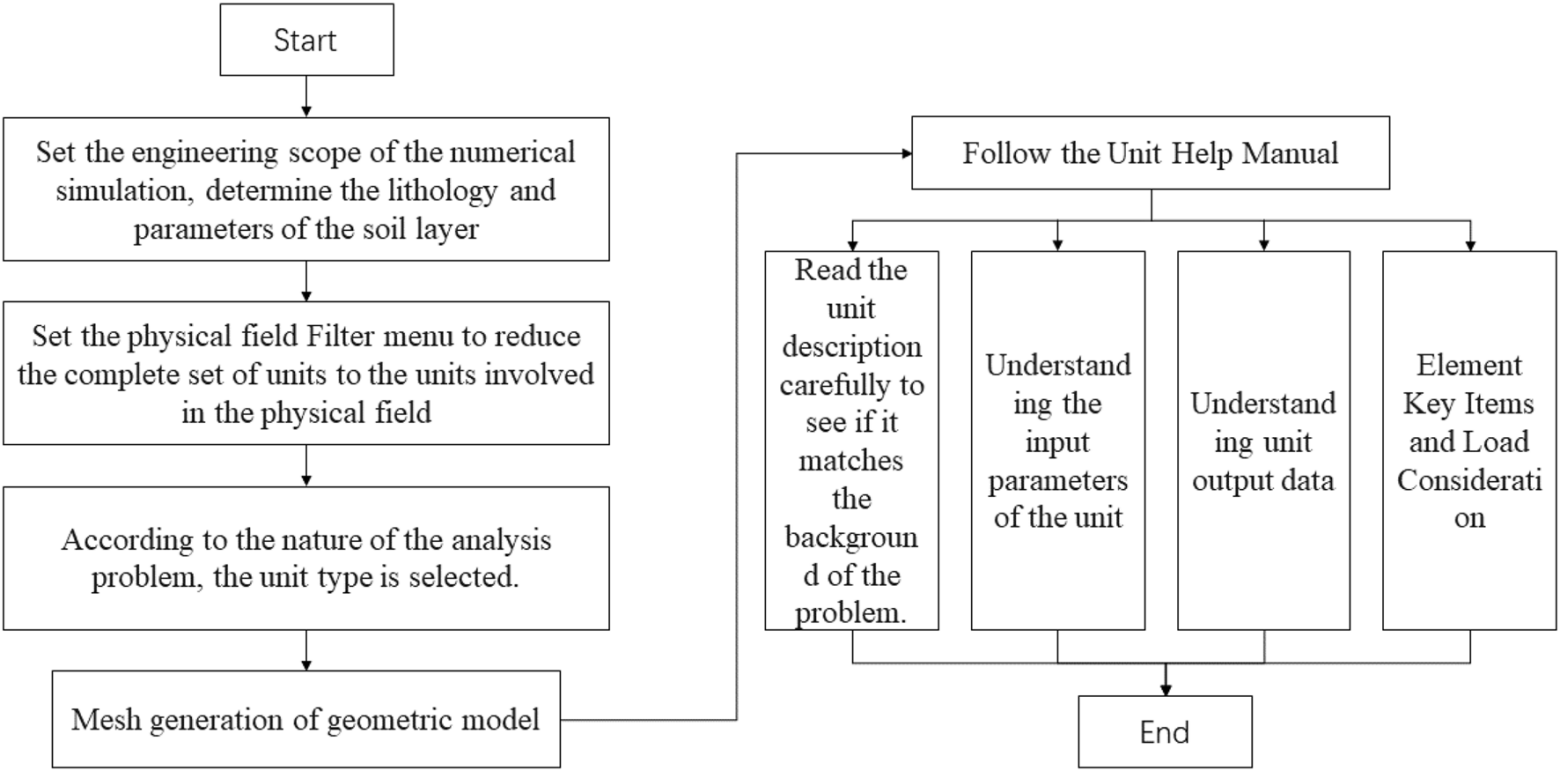
Modeling stages process of Lijiazhai slope numerical model.

The process flowchart of the Numerical modeling stages is shown in Fig. 5.

Abaqus software was used in the numerical simulation. The simulated region was 420 m along the longitudinal road in the forward direction and 400 m in the horizontal and vertical directions. The highest elevation was approximately 336 m, whereas the lowest elevation was approximately 213 m.

The thickness of the strongly weathered layer was uneven. The thickness of the ditches and valleys generally exceeded 15 m, whereas that of the thin ridge zone was less than 5 m. The classification of the fully weathered rock mass belongs to surrounding rock class V^35^, whereas the slightly weathered rock mass belongs to surrounding rock Class IV, as shown in Table 5.

**Table 5:** Rock parameters of Lijiazhai slope.

Palimpsest siltstone	Gravity density (kN/m^3^)	Poisson’s ratio	Deformation modulus (GPa)	Cohesion (MPa)	Friction angle (°)
Full-strong weathered	19	0.40	3.1	0.12	24
Slightly weathered	22	0.32	3.5	0.40	34

**Figure 6:**
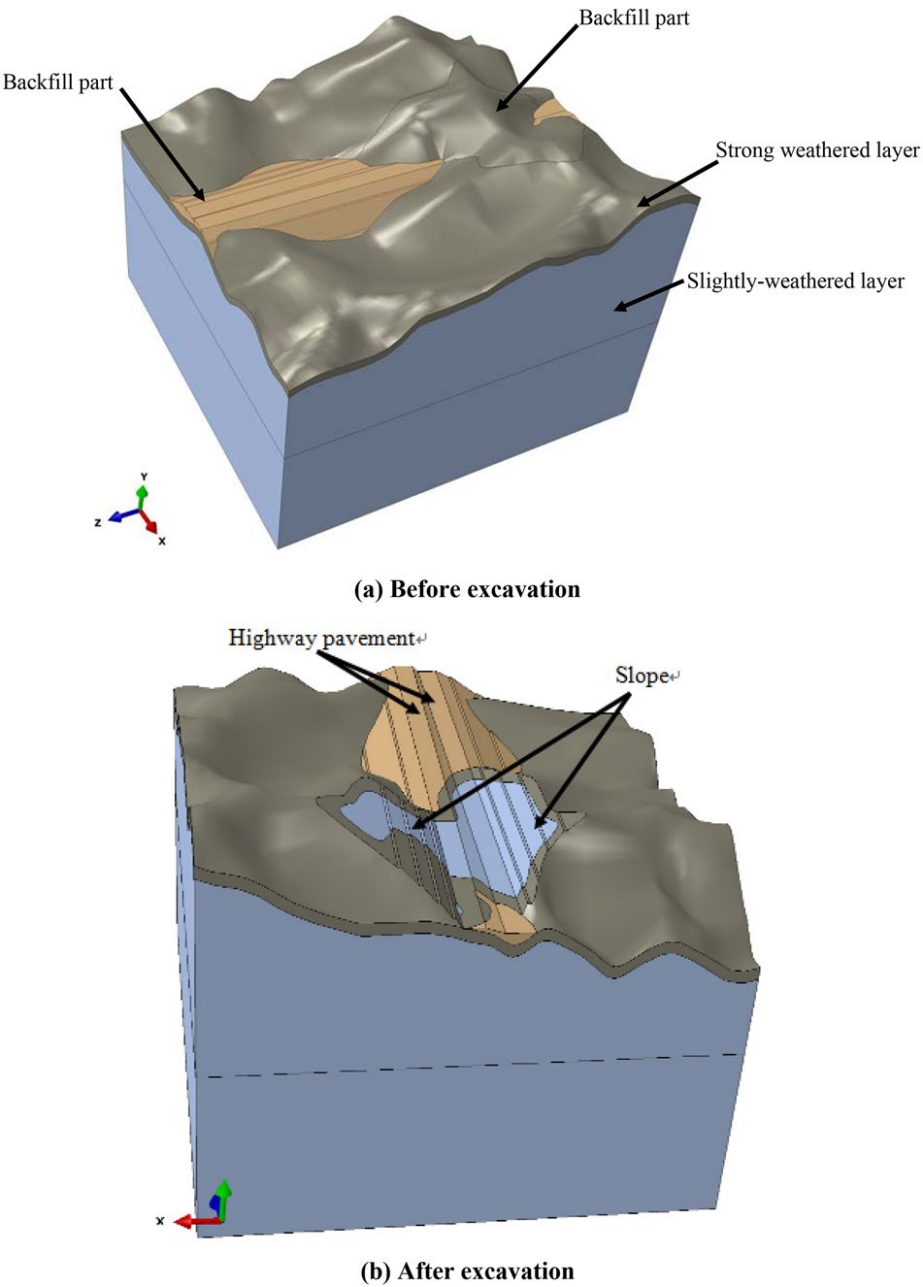
3D model of the Lijiazhai slope before and after excavation.

The strongly weathered layer and slightly weathered layer, as well as the backfill and excavation parts of these entities, are shown in Fig. 6.

#### Parameters

The parameters of the Mohr–Coulomb elastic–plastic model included elastic modulus, Poisson's ratio, cohesion, and internal friction angle (Table 4).

#### Element type

**Figure 7:**
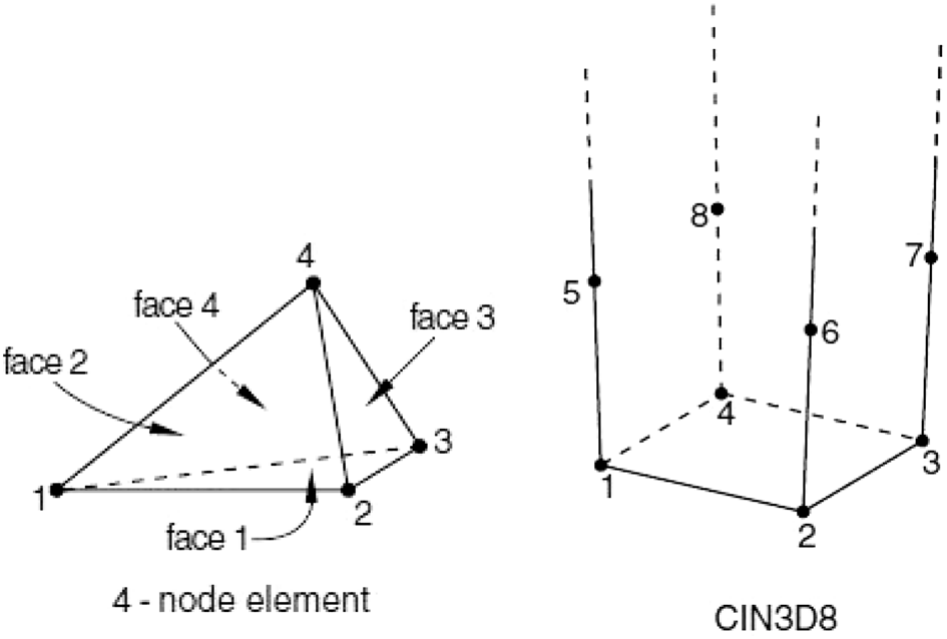
Adopted element type in numerical simulation.

Given that the topographic surface of the model was complex, linear tetrahedral units were adopted in the upper part of the model. Given that the effect of the strata on the model within 150 m of the bottom layer was small, the infinite element was used (Fig. 7). The displacement was determined by linear interpolation. The interface displacement at the bottom edge was zero, and the contact displacement between the infinite and finite elements was calculated using the result of the calculation of the finite element.

**Figure 8:**
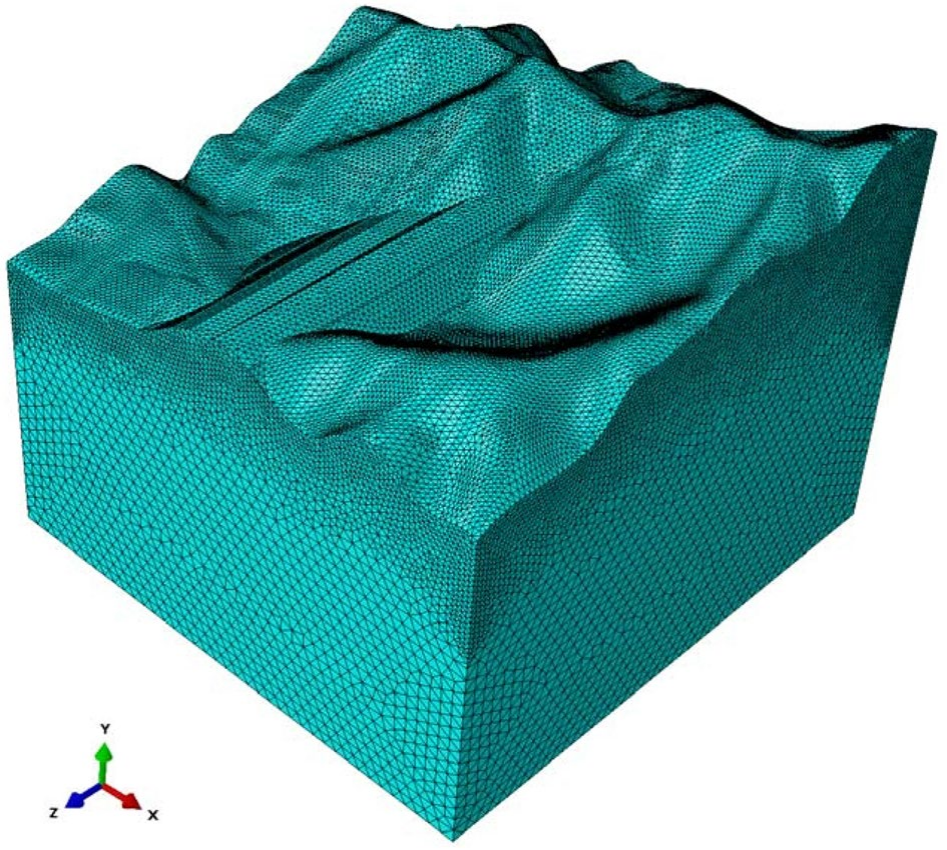
Grid of FEM model of Lijiazhai slope.

The principle of encryption from the bottom was adopted in the model units (Fig. 8). The bottom of the ground changed from 40 to 10 m, the center changed from 10 to 4 m, and the upper part changed from 4 to 2 m. The local details were 1 m. A total of 347,023 units were calculated. The retaining structures were not considered in the simulations.

## Results and discussions

### 3D surface deformation trend

**Figure 9:**
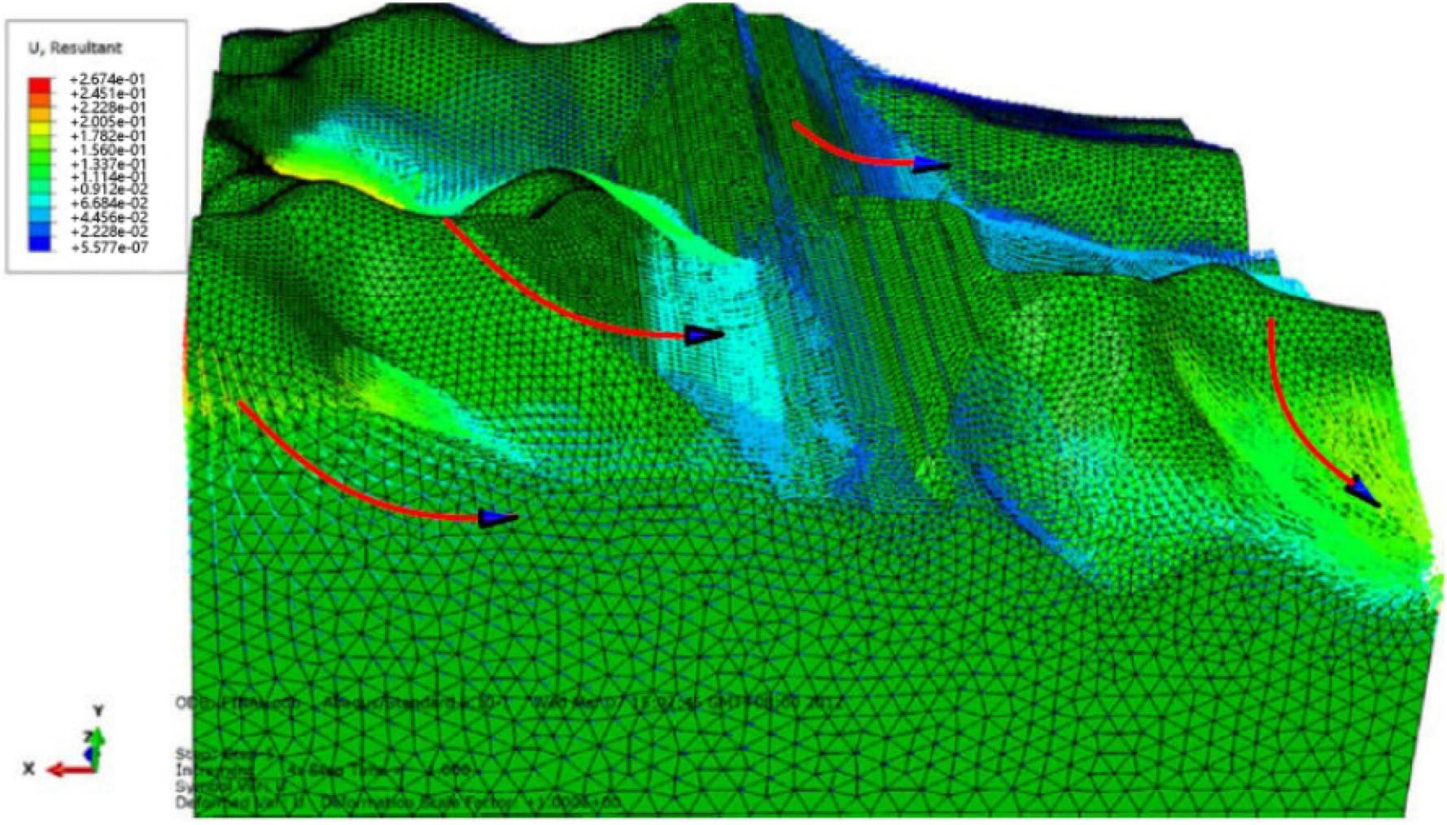
Potential slip trend of Lijiazhai slope incicating displacement**.**

The 3D characteristics of slope failure and deformation are obtained from the total displacement trends within the scope of the entire simulation, and the potential slip part of the slope is marked according to the total displacement trend (Fig. 9).

**Figure 10:**
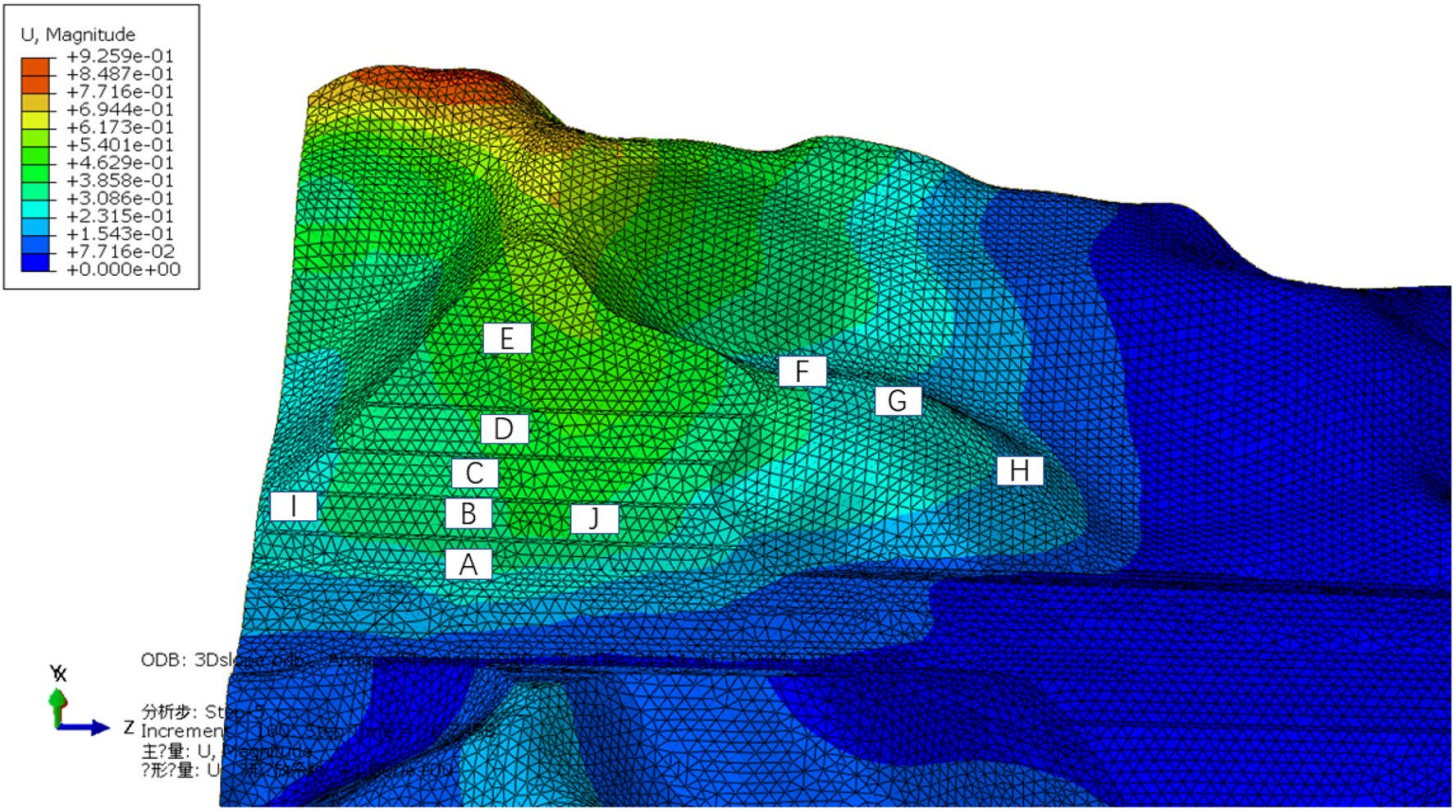
Monitoring points in the numerical simulation of the Lijiazhai slope.

**Figure 11:**
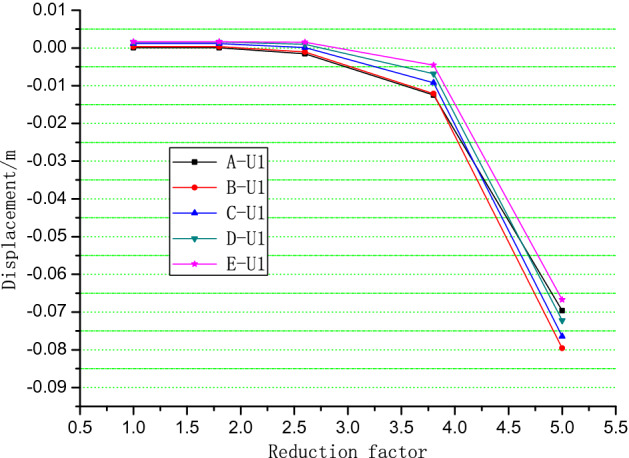
Displacement of monitoring points A to E vs. reduction factor.

**Figure 12:**
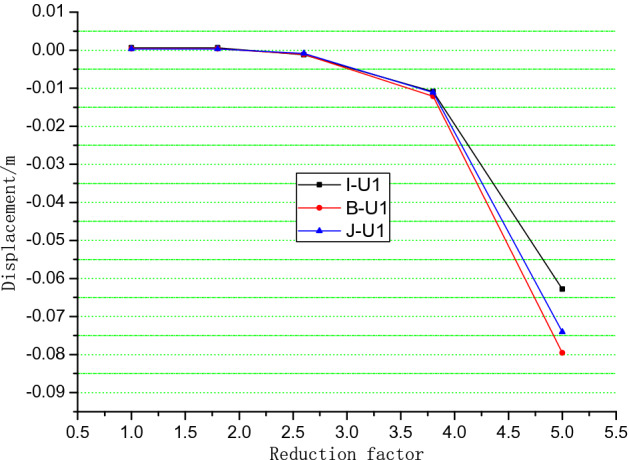
Displacement of monitoring points I-B-J vs. reduction factor in the x direction.

For the total deformation trends analysis, the simulation results of different positions are shown in Figs. 10, 11 and 12. When the reduction factor became larger, the displacement along the Z axis increases as elevation increased. Monitoring the displacement of Point A was important because it was close to the potential shear exports of a landslide. The displacement of monitoring Points B, I, and J in the X direction indicates that the displacement of intermediate Point B was the largest and that the other two sides were small. This finding verified that the potential slip surface was characterized by an arc shape.

**Figure 13:**
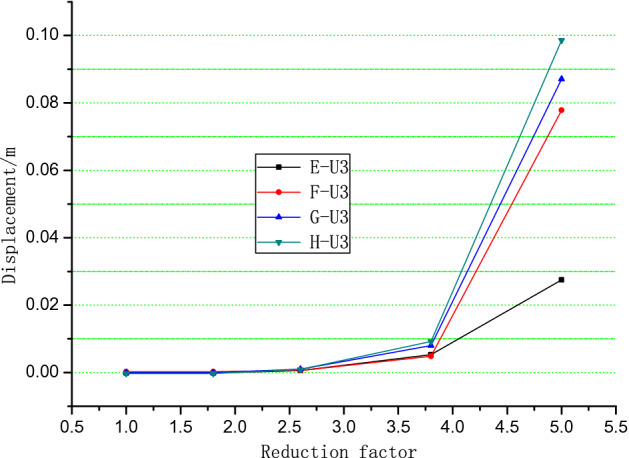
Displacement of monitoring points E to H vs. reduction factor in the z direction.

The slope deformed in the direction perpendicular to slope orientation (Fig. 13), causing the potential slip direction of the slope to deviate from the slope orientation. The deformations of Points F, G, and H indicate that the deformation of the slope ridge manifested 3D features.

When the reduction factor was between 1.0 and 2.5, the displacement of the slope was very small, indicating that the slope was in a steady state. The displacement increases when the reduction coefficient exceeded 2.5. When the reduction factor reached 5.0, the displacement was equal to 0.08 m, which indicates that the slope was in the failure stage (Fig. 13).

### Initial position of failure

**Figure 14:**
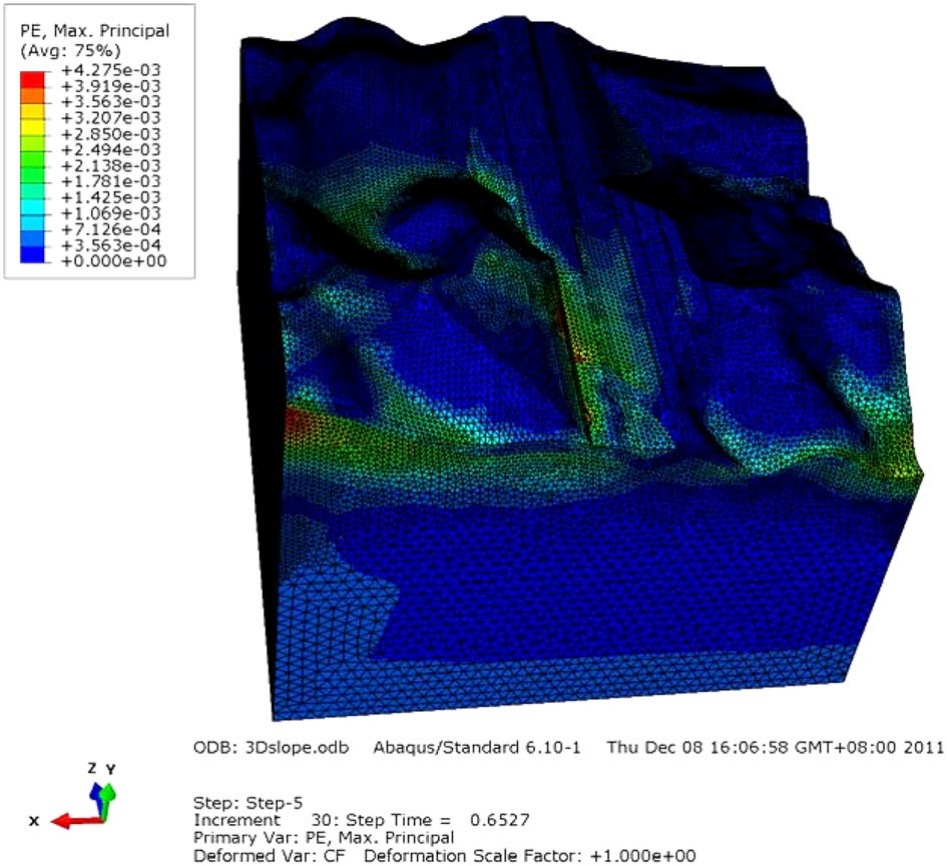
Contour of equivalent plastic strain under SRM.

The simulated plastic strain is shown in Fig. 14. The starting point of failure of the Lijiazhai slope was located at the position with larger plastic strain, mainly in the slope trailing edge and foot.

**Figure 15:**
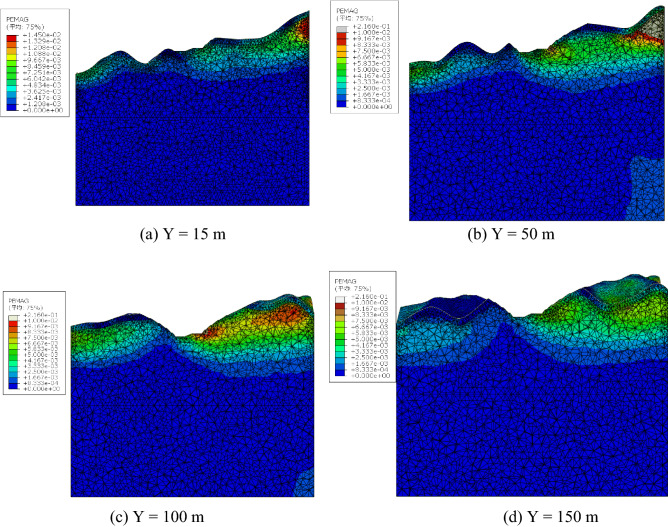
Contour of equivalent plastic strain.

**Figure 16:**
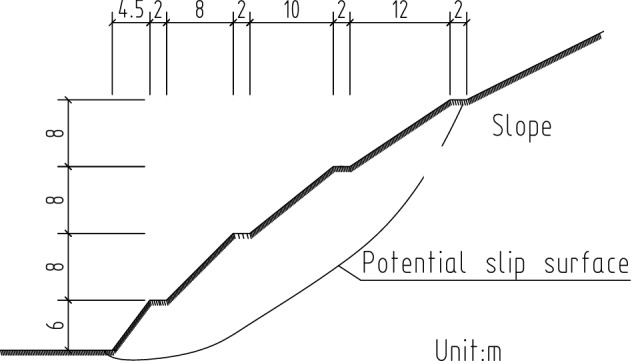
Position of potential slip surface at the top and foot of the slope.

To determine the internal development of the plastic zone, the slope within the cross-section along the Y direction was analyzed (Fig. 15). Plastic strain appeared at the right side of Fig. 15 at the foot of the slope. The plastic zone was narrow at both sides along the slope direction, intermediately wide, substantially arcuate, and concentrated in the vicinity of the slope foot, extending but not penetrating. The quantitative results indicated the obvious deformation zone and plastic area. In numerical simulations, the occurrence of a plastic zone means that the part has yielded under current stress. However, a local plastic zone means local yield. The superficial failure means the start of local failure instead of an unstable slope. The penetration of the plastic zone results in instability. The plastic zones indicated the local failure of rock mass and the start of the landslide course although the slope was stable. The theoretical slip surface on the slope foot and top are shown in Fig. 16.

**Figure 17:**
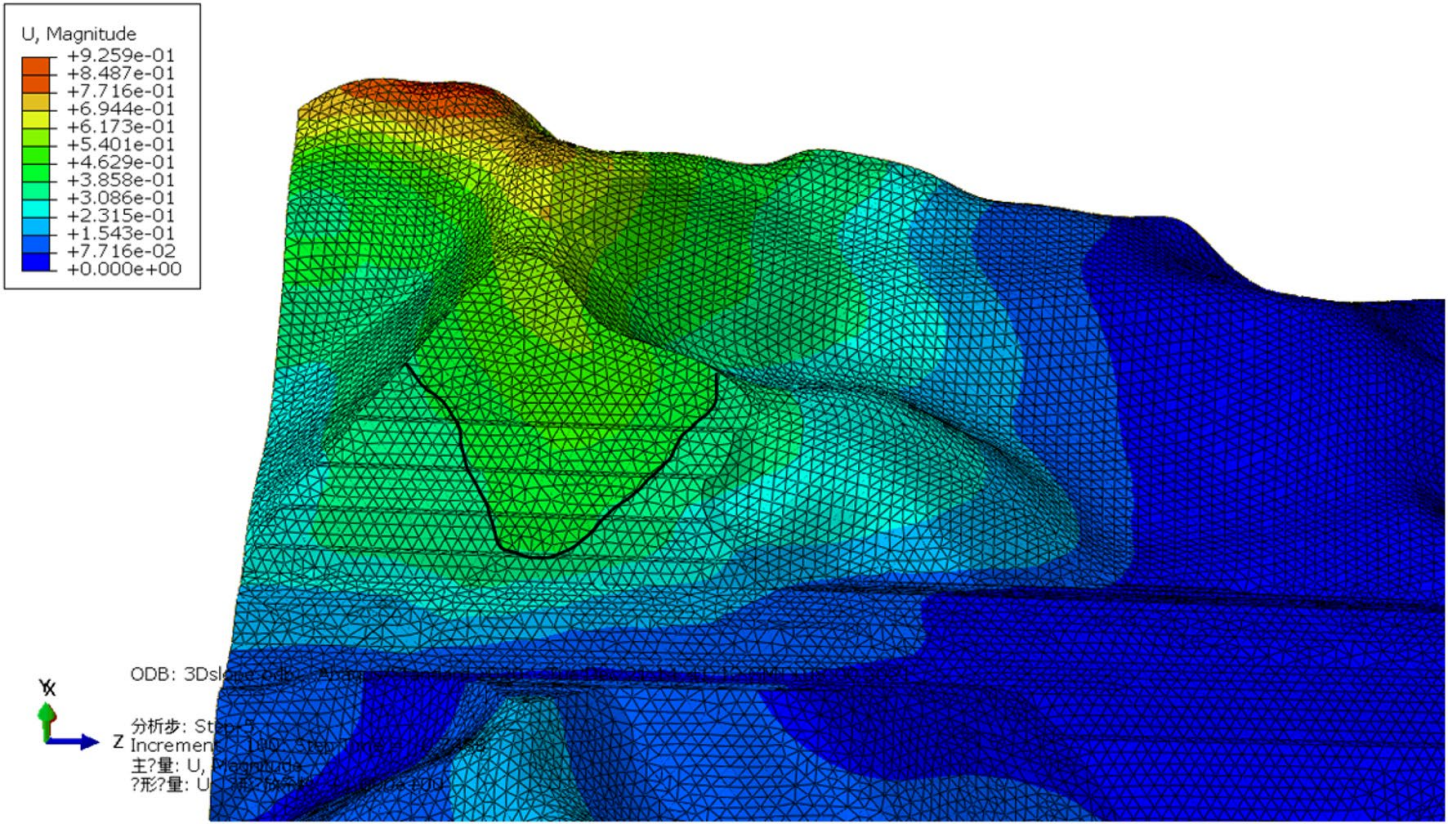
Potential slip surface determined by the equivalent plastic strain.

In Fig. 17, the boundary of certain equivalent plastic strain that penetrates the slope can be defined as the potential sliding surface.

### Maximum depth of potential slip surface

**Figure 18:**
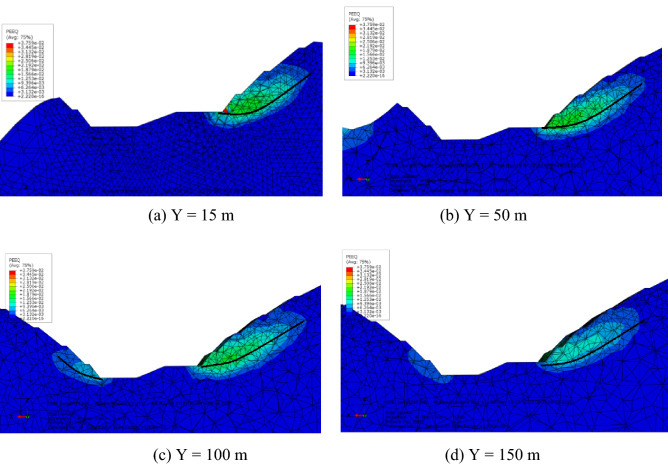
Contour of equivalent plastic strain along the Y direction.

**Figure 19:**
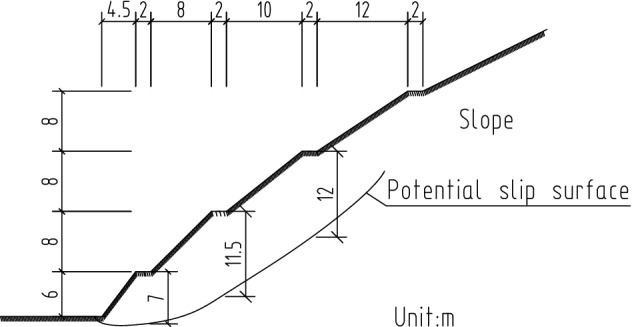
Depth of potential slip surface of Lijiazhai slope.

Plastic strain occurred at the bottom and extended to the third platform of the slope. The potential slip surface of the slope occurred in the area with a larger plastic strain. Cross sections of equivalent plastic strain contours can clearly identify the location of the potential slip surface (Figs. 17 and 18). The vertical distances of the three platforms from the potential slip surface were 7.0, 11.5 and 12.0 m. The location of the potential failure surface is shown in Fig. 19.

## Optimization of slope monitoring based on 3D slope deformation and failure characteristics

### Displacement monitoring network arrangement scheme

**Figure 20:**
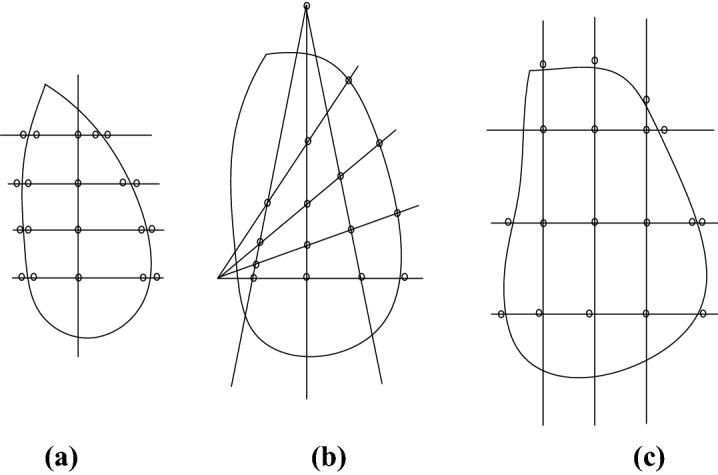
Normal surface displacement monitoring network.

The displacement monitoring networks can be arranged based on experience and previous studies in monitoring design. For a long and narrow landslide with limited scope and significant spindle, a criss-cross monitoring network should be arranged (Fig. 20a). For a landslide with limited scope as well as a wide and open terrain, a radiation monitoring network should be arranged (Fig. 20b). For a large landslide with complex terrain, an arbitrary square grid monitoring network should be adopted (Fig. 20c).

Cross, radiation, or grid-based monitoring networks can be arranged when the landslide perimeter and potential slip surface have been determined based on an onsite investigation. However, the 3D deformation and failure features are not considered. If a large number of monitoring points are arranged in a stable area, monitoring cannot provide valid data on slope stability, which is wasteful. Moreover, insufficient monitoring points may be arranged in the unstable part where the monitoring of the sensitive point for slope stability cannot provide timely warning, in turn potentially causing accidents or losses.

A monitoring network arrangement method based on 3D slope deformation and failure characteristics was proposed. The overall concepts and steps were as follows. (1) Experience inference on the deformation and failure mode of a slope was made based on investigation data and reasonable analysis of geological structure. (2) 3D numerical models were established to verify numerical simulation results. (3) 3D numerical simulation using SRM was performed to analyze the stability reserve of the slope, the 3D deformation and failure characteristics of the slope, and the extent of deformation and failure analysis. (4) Hazardous areas or sections were marked according to the total displacement trend and potential sliding zone. (5) Monitoring point arrangement was suggested based on the plane and depth direction to provide a quantitative basis for monitoring, as well as protection based on 3D deformation and failure characteristics. (6) Monitoring arrangement was optimized.

### 3D surface displacement monitoring

#### Slope displacement partition on plane

**Figure 21:**
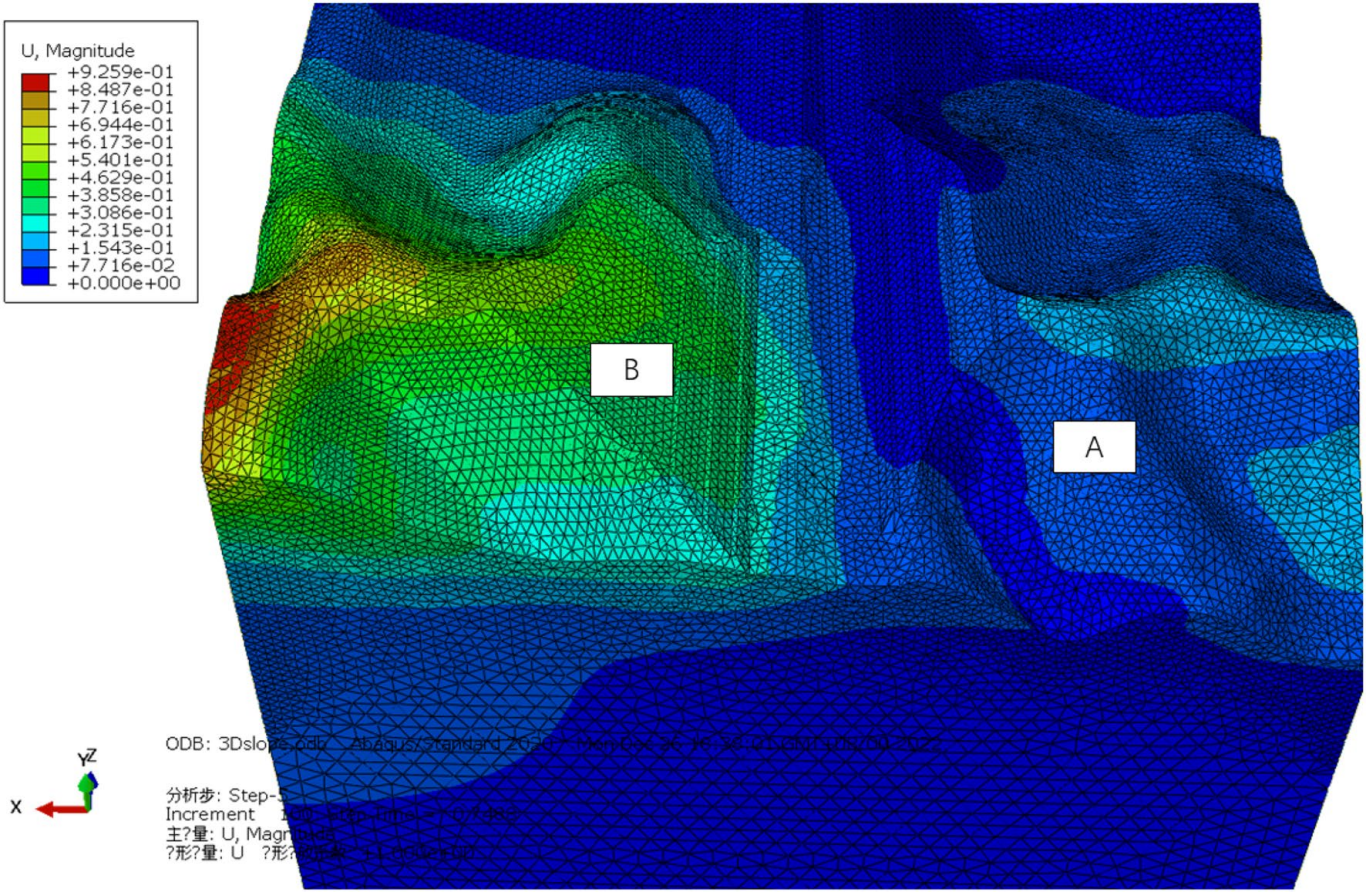
Total displacement of the slope.

The area with a larger displacement can be determined by using 3D numerical simulation (Fig. 21). The surface deformation of Slope B was large and increased with the increasing elevation, whereas the surface deformation of the right slope was small and decreased as the elevation increased.

**Figure 22:**
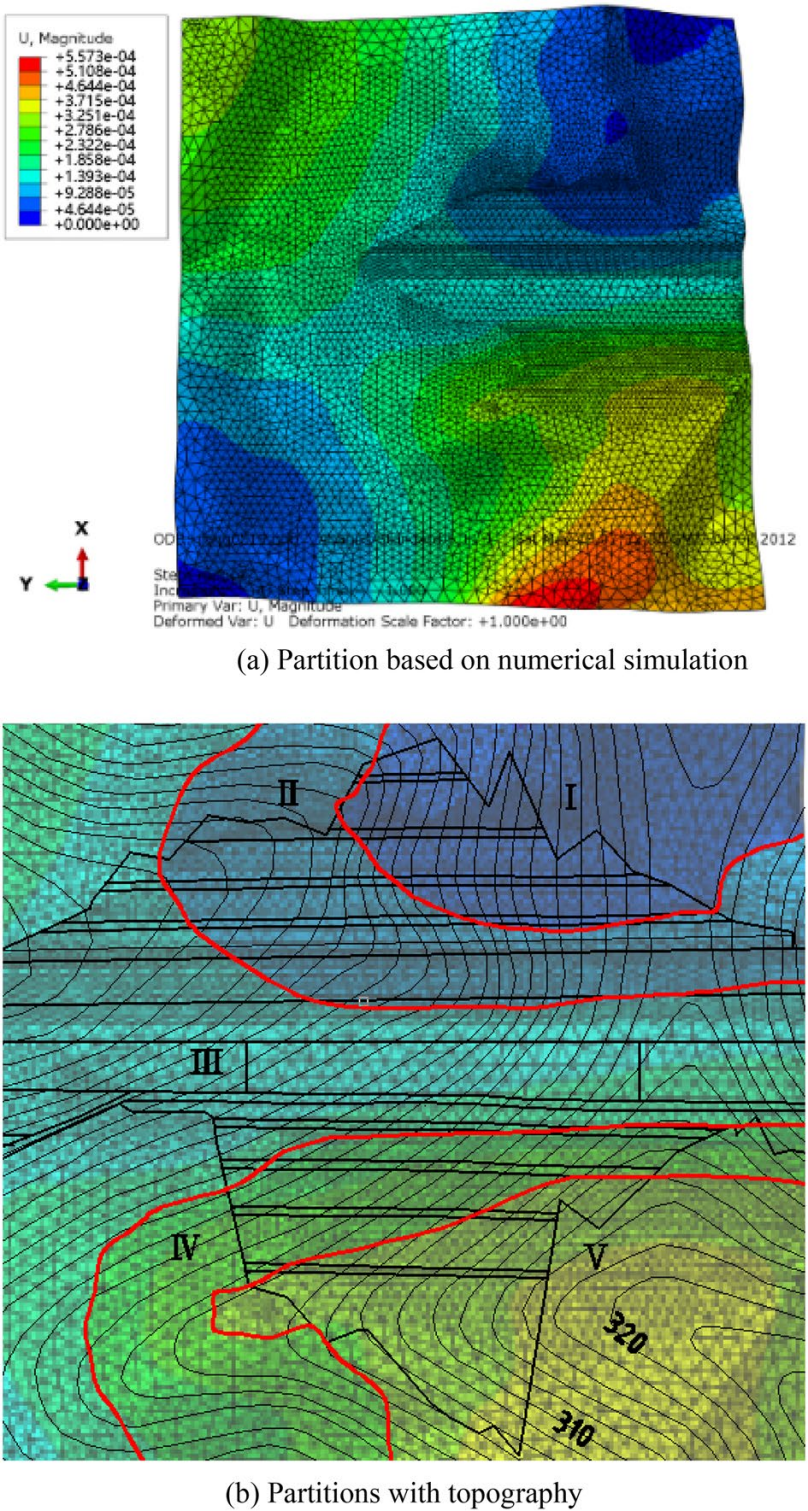
Monitoring partition based on displacement.

Based on the magnitude of the displacement, the slope was divided into five regions (Fig. 22):

- Region I: Displacement U was approximately zero;
- Region II: Displacement U = 0 cm to 1 cm;
- Region III: Displacement U = 1 cm to 2 cm;
- Region IV: Displacement U = 2 cm to 3 cm;
- Region V: Displacement U > 3 cm.

The deformation of Slope A was generally small. The range from the third platform to the slope top was located in Region I, where the deformation was approximately zero. The deformation of Slope B generally was larger than 2 cm in the range from the first three slope platforms to the top of the slope. The deformation in this slope trailing edge was larger than 5 cm. Therefore, the surface displacement monitoring points should be arranged in Region V.

**Figure 23:**
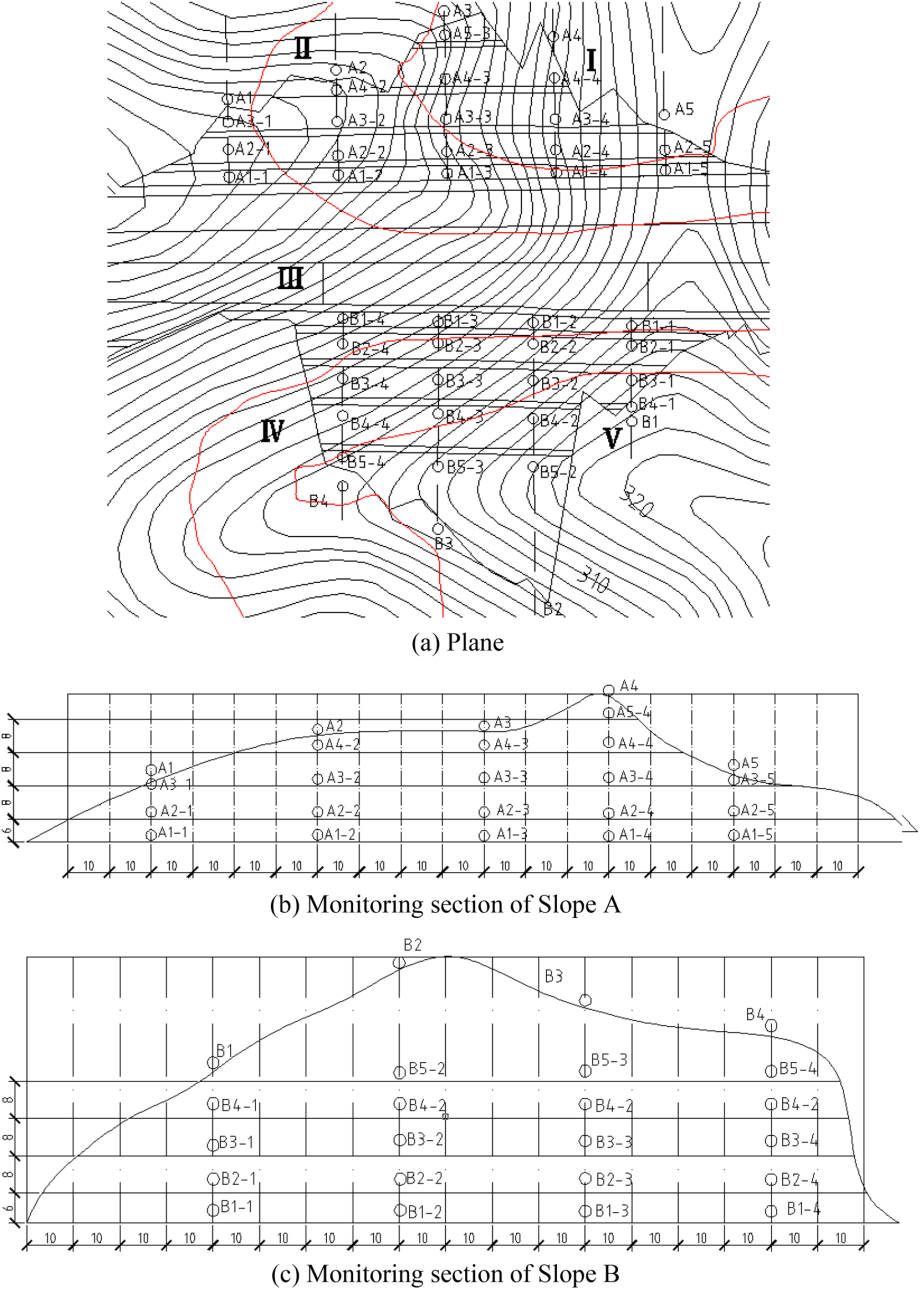
Monitoring arrangement based on specification.

The monitoring networks designed according to corresponding specifications are shown in Fig. 23. Monitoring points were originally arranged within the box-shaped grid layout. The horizontal spacing of monitoring points on the platform at all levels was 40 m.

Based on 3D simulation, the deformation above the third platform to the top of Slope A was approximately zero. Thus, the monitoring points should be adequately reduced. The deformation of Slope B, particularly on the fifth platform and at the end edge, exceeded 5 cm. Thus, the monitoring points should be encrypted.

**Figure 24:**
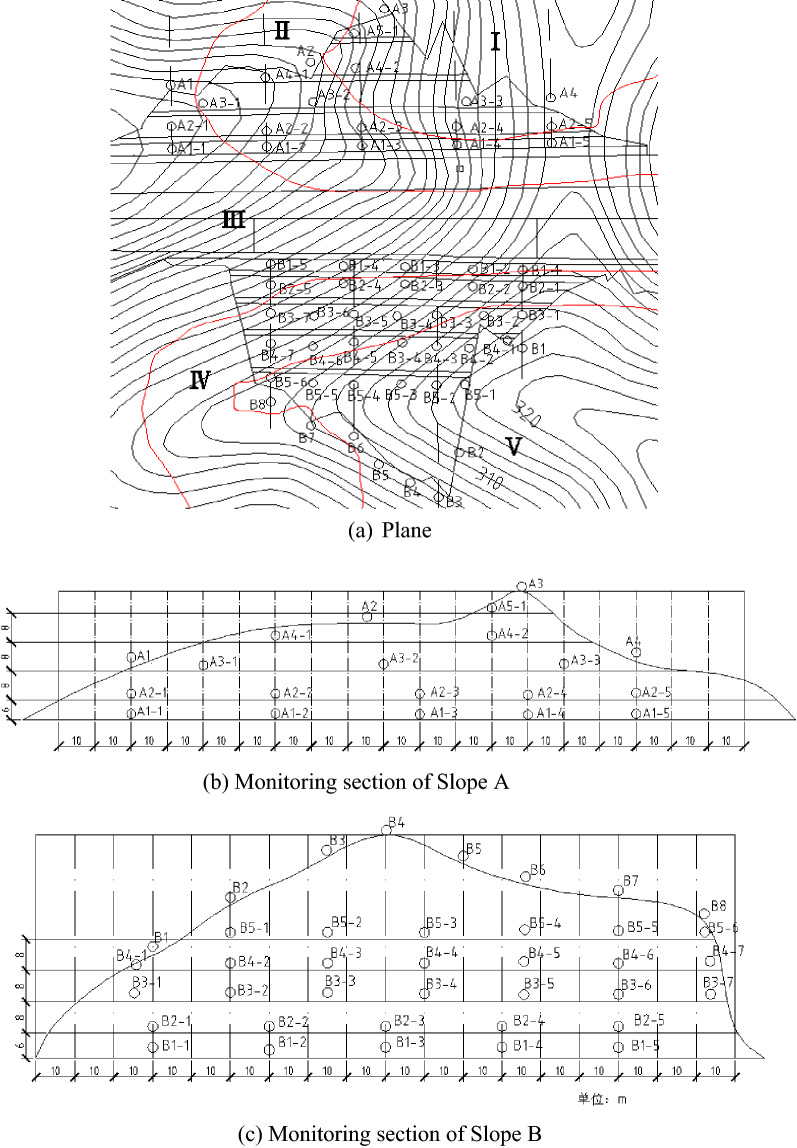
Optimized monitoring arrangement considering 3D characteristics.

The optimized monitoring network is shown in Fig. 24. The surface displacement monitoring points of Slope A were arranged on the platform. The horizontal spacing for the first two platforms was 40 m. Given that the deformations of the third to the fifth platforms were small, the monitoring points can be appropriately reduced and the horizontal spacing should be 50 m.

The overall surface deformation of Slope B was large, and monitoring points should be encrypted. Each platform had a minimum of five monitoring points. The horizontal spacing of monitoring points between the first two platforms was 30 m, and that of the third to the fifth platforms was 25 m.

#### Deep displacement monitoring

Determining the internal displacement monitoring position of a slope as well as analyzing the 3D features of the internal process of slope failure using model tests and field monitoring methods was difficult. Thus, the 3D numerical simulation method can be used to analyze the displacement direction within the cross-section. In the numerical simulations, SRM was performed to simulate the influence of natural factors on slope stability.

**Figure 25:**
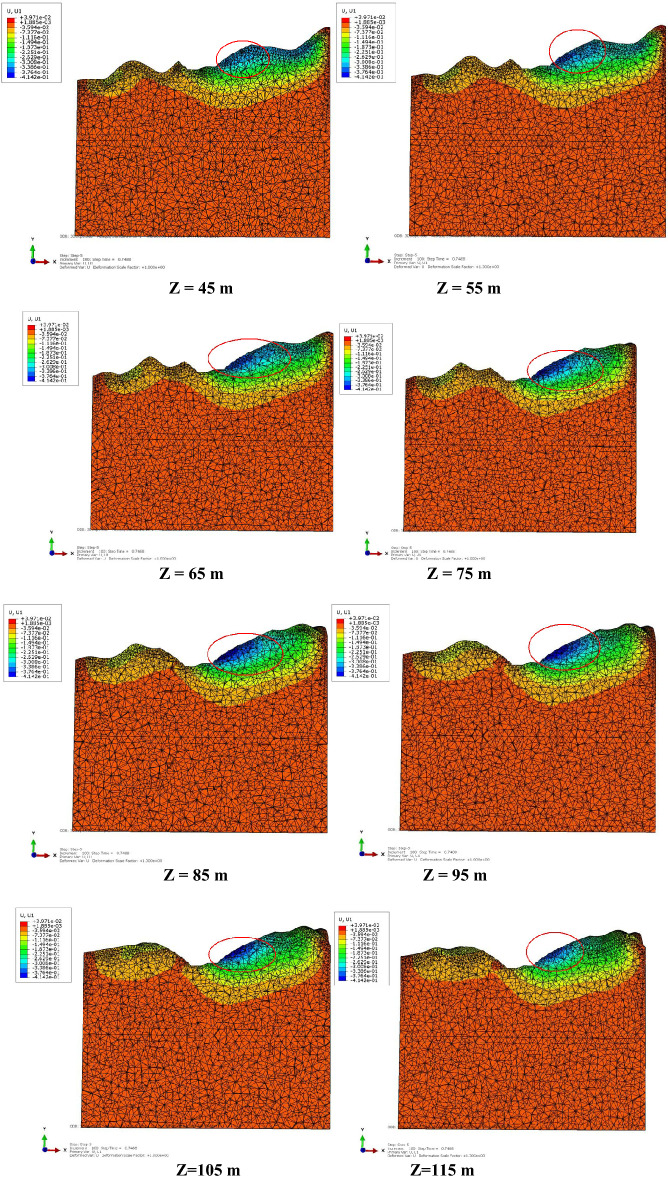
Displacement in the x direction between 45 and 115 m.

To determine the displacement of a slope using the 3D model, analyzing the deformation in the cross-section of the slope with internal displacement contours corresponding to the SRM was necessary (Fig. 25). The position within the red circle was the excavation area. The cross-section in the Z direction within the range of 45 m to 115 m is shown for comparison.

The area with a larger displacement first increased and then decreased as the Z direction extended when the elevation of Z was between 75 and 85 m. For shallow displacement, the depth of displacement monitoring holes can also be reduced correspondingly. For displacements in deep locations, adequate drilling depth of monitoring holes should be designed to ensure effective and accurate monitoring.

**Figure 26:**
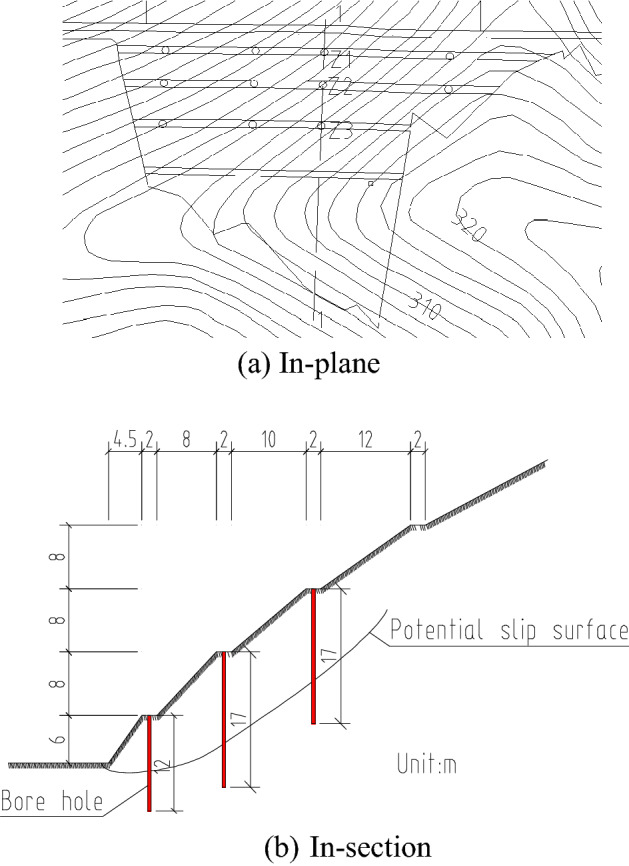
Arrangement of deep displacement monitoring.

Deep monitoring points were arranged on the slope platform at all levels. The spacing was 40 m, and the number of monitoring points was less than three at each level. The theoretical position of the potential failure surface was obtained based on previous 3D simulations and engineering survey specifications^35,36,38,39^. The depth of the displacement monitoring device installed in a borehole should run through and cross the position of the potential sliding surface. The bottom of the borehole should be 5 m deeper than the predicted potential sliding surface. Thus, the drilling depth of the borehole on the first level of the slope platform should be 12 m, and that of the second and third levels should be 17 m. Deep monitoring points were not arranged on the fourth level of the slope platform (Fig. 26).

## Discussions

The potential slide mode of an engineering slope was mainly determined by the natural geological structure and human excavation. Normally, the stability of a natural slope can sometimes be determined through analogy with historical landslides. Monitoring design can be performed by geological analysis and experience. However, the 3D characteristic of an engineering slope cannot be understood or identified only by engineering analysis through experience. 3D numerical analysis can provide a reference for the stability analysis of an engineering slope, including monitoring and excavation optimization.

During the operation stage, the stability of an engineering slope may be influenced by multiple factors, including rainfall and anthropic factors, due to the increase in anthropic factors and the reduction of strength. In the operation stage, the excavation and loading of engineering were not the main influencing factors. However, the decreasing rock mass strength, which was disturbed during the construction stage, became the main influencing factor. Under the vibration and vehicle load, the weathering through the new excavation face and cracks, together with the influence of rainfall and groundwater, may decrease the long-term strength of rock mass. Thus, the reducing rock mass strength indicated by the SRM was considered here.

Given that only elastic–plastic characteristics were considered in the present study, the displacement cumulative effect was not considered here. The displacement trend was considered to recognize the extremely adverse mechanical conditions during the operation stage. The displacement cumulative effect with time should be considered in future research.

The SRM methods can provide quantitative references for the optimization of slopes in operation. The monitoring points can be arranged in the key position with disadvantageous stress and displacement conditions. With the development of innovative calculation methods, new SRM-based methods can be introduced and developed. The new SRM-based methods can be combined with random influencing factors to predict unfavorable scenarios. The SRM-based method can produce multi-scenario data, which can be used to recognize the non-linear potential relation between potential slide surfaces and SRM.

The purpose of the current SRM numerical simulation was to obtain the 3D characteristic of an operation slope instead of precise numerical simulations, so the parameters were selected according to geotechnical investigation. The verifications were confirmed by the correct parameters from the investigation. In future research, back-analysis should be performed to determine the parameters precisely and verify the simulation results in detail.

## Conclusions

1. SRM was adopted in the 3D numerical simulation of the Lijiazhai slope. The results show that the potential slope deformation and failure clearly involve 3D features. The 3D slope deformation and failure characteristics should be considered in the slope monitoring network arrangement.
2. The potential boundaries of sliding and stable areas were obtained using 3D numerical simulation with SRM, providing a theoretical basis for the surface monitoring network density layout and the depth of the underground monitoring network arrangement.
3. Potential shear export position and potential slip surface locations can provide a reference for the monitoring arrangement and depth in plane based on a focused monitoring program.
4. The results can provide references for 3D slope stability analysis and monitoring during the operation stage.

## Data Availability

All data generated or analysed during this study are included in this published article.
